# Novel cardioprotective and regenerative therapies in acute myocardial infarction: a review of recent and ongoing clinical trials

**DOI:** 10.2217/fca-2016-0044

**Published:** 2016-10-28

**Authors:** Nicholas B Spath, Nicholas L Mills, Nicholas L Cruden

**Affiliations:** 1 Centre for Cardiovascular Science, University of Edinburgh, Edinburgh, UK; 2 Edinburgh Heart Centre, Royal Infirmary of Edinburgh, Edinburgh, UK

**Keywords:** cooling, ischemic conditioning, ischemia-reperfusion injury, myocardial infarction, pharmacological conditioning, primary percutaneous coronary intervention, stem-cell therapy

## Abstract

Following the original large-scale randomized trials of aspirin and β-blockade, there have been a number of major advances in pharmacological and mechanical treatments for acute myocardial infarction. Despite this progress, myocardial infarction remains a major global cause of mortality and morbidity, driving a quest for novel treatments in this area. As the understanding of mitochondrial dynamics and the pathophysiology of reperfusion injury has evolved, the last three decades have seen advances in ischemic conditioning, pharmacological and metabolic cardioprotection, as well as biological and stem-cell therapies. The aim of this review is to provide a synopsis of adjunctive cardioprotective and regenerative therapies currently undergoing or entering early clinical trials in the treatment of patients with acute myocardial infarction.

Ischemic heart disease remains a leading cause of death worldwide, responsible for an estimated 17.5 million deaths in 2012 [1]. The persistence of ischemic heart disease as a major cause of global mortality and morbidity despite major therapeutic advances in this area, along with an evolving understanding of the reperfusion injury itself, has led to a focus on novel therapies to limit myocardial injury and facilitate myocardial recovery following acute myocardial infarction. With numerous strategies proposed, this review will provide a synopsis of novel therapies currently in research development or undergoing clinical trials, in addition to considering future directions for exploration.

## Current treatments for acute myocardial infarction

**Figure 1.**
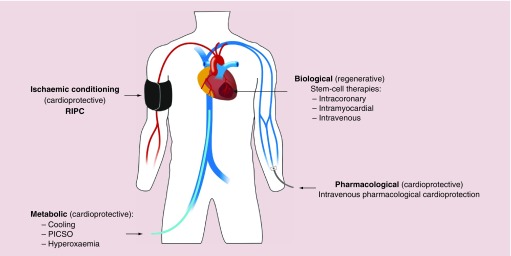
**Overview of novel therapeutic strategies.** RIPC: Remote ischemic preconditioning; PICSO: Pressure-controlled intermittent coronary sinus occlusion.

The current management of patients presenting with acute myocardial infarction focuses on antiplatelet and antithrombotic treatment coupled with invasive assessment of coronary anatomy with a view to revascularization where appropriate [2–4]. Pharmacological therapies such as β-blockade, statins and inhibitors of the renin–angiotensin–aldosterone axis improve cardiac remodeling and subsequent cardiac events including myocardial ischemia and the development of heart failure [5]. Despite these advances and the establishment of clinical standards and national campaigns to promote guideline-driven management of patients with acute myocardial infarction, ischemic heart disease remains a major global cause of death and disability. The search for more effective treatments to limit myocardial injury following abrupt coronary artery occlusion continues. Major areas of endeavor include the prevention of reperfusion injury by mechanical or pharmacological means, regenerative therapies for the myocardium and metabolic strategies including cooling (Figure 1). This review will summarize major progress in these areas to date.

## Methods

A literature search was performed by two authors using PubMed and ClinicalTrials.gov using the following key words: ‘novel therapies’, ‘regenerative therapies’, ‘myocardial infarction’, ‘reperfusion injury’, ‘ischemic conditioning’, ‘pharmacological conditioning’ and ‘cardioprotection’ to identify relevant ongoing or recent clinical trials and systematic reviews on cardioprotective and regenerative therapies for acute myocardial infarction undergoing current or recent clinical trial [6]. We limited our search to papers published between 1950 and 30 April 2016. Any papers identified through the bibliographies of the original articles were subsequently screened.

## Reperfusion injury & ischemic preconditioning

While restoration of myocardial perfusion remains the cornerstone of treatment for acute coronary occlusion, reducing the insult caused by the ensuing reperfusion injury has attracted significant interest in recent years. Myocardial reperfusion injury describes the damage to or death of vulnerable myocytes that occurs as a result of restoration of blood flow and has been seen following thrombolysis, percutaneous coronary intervention (PCI), coronary artery bypass grafting and cardiac transplantation [7]. Central to the pathophysiology of reperfusion injury is the mitochondrion, where overproduction of reactive oxygen species (driven further by neutrophil activation) and dysfunctional calcium handling leads to altered myocardial metabolism as well as microvascular and endothelial dysfunction [1,8–9].

## Mitochondrial dynamics in cell death

Our understanding of mitochondrial dynamics has advanced significantly in recent years, notably of their role in the regulation of cell death. Independent of mode, cell death is intrinsically linked to mitochondrial dynamics through regulation of mitochondrial membrane permeabilization which, once induced, initiates a rapid and irreversible cascade of enzymatic interactions that ultimately leads to organized cell destruction [10]. Mitochondrial membrane permeabilization is dependent on a complex interplay between numerous regulatory mechanisms at mitochondrial membranes; the proposed mechanism by which remote ischemia conditioning prevents opening of the mitochondrial permeability transition pore (mPTP) within the inner mitochondrial membrane [11]. Recognition of mitochondrial membrane permeabilization as the decisive event in myocardial cell death by ischemia reperfusion injury [12], in addition to most pathophysiology of cell death, has led to inhibition of this process becoming a major therapeutic target in this field.

## Cardioprotective therapies

### • Ischemia conditioning

#### Ischemic preconditioning

The phenomenon of ischemic preconditioning, administration of periods of short-lived ischemia prior to a more prolonged ischemic insult, was first demonstrated almost 30 years ago by Murry *et al*. [12] who observed a dramatic reduction in infarct size in canine hearts following administration of local, transient myocardial ischemia prior to total occlusion of the circumflex artery. Ischemic preconditioning (IPC) has subsequently been shown to have a beneficial effect in animal and human studies, in the heart as well as other organs [13]. The preconditioning stimulus can be delivered either locally or remotely to the target organ at risk of infarction with numerous molecular signaling pathways implicated, although the exact mechanisms have yet to be established [14,15].

The invasive nature and temporal requirements of the local preconditioning stimulus relative to the ischemia-reperfusion insult have limited the applicability of local IPC in the setting of acute myocardial infarction. Consequently, attention has focused on remote IPC (rIPC), where tissues other than the heart are exposed to ischemia protocols, with subsequent signaling pathways resulting in a reduction in myocardial reperfusion injury. This phenomenon was first demonstrated within the canine coronary arterial tree where transient occlusion of circumflex artery prior to induction of ischemia reperfusion injury in the left anterior descending artery in canine models resulted in a significant reduction in infarct size (6 vs 16% in controls) [16]. The most convincing evidence of a benefit with IPC in man arises from trials in patients undergoing cardiac surgery. A recent meta-analysis of IPC in patients undergoing cardiac surgery included 22 eligible trials with a total of 933 patients [17]. In the majority of studies, the IPC stimulus consisted of cycles of brief aortic cross-clamping, although three trials utilized coronary artery occlusion. Overall, IPC was associated with a significant reduction in ventricular arrhythmia, inotropic requirement and length of stay in intensive care unit [17].

For obvious reasons, cross-clamping of the aorta would be impractical in patients presenting with acute myocardial infarction. As a surrogate, transient limb ischemia induced by inflation of a blood pressure cuff to suprasystolic pressure has also been shown to be sufficient to induce myocardial protection in patients exposed to ischemia-reperfusion injury. Three recent meta-analyses of rIPC using transient limb ischemia as the stimulus [18–20] (including five studies [n = 731], nine studies [n = 1119] and 11 studies [n = 1713], respectively) confirmed an observed reduction in procedural cardiac biomarker release in patients undergoing predominantly elective PCI with rIPC compared with control. It remains unclear whether a reduction in cardiac marker release will translate into a clinically significant reduction in major adverse cardiac and cerebrovascular events (MACCE) [21]. These meta-analyses were published prior to the publication of the large clinical outcome ERICCA trial [22] that included 1612 patients undergoing coronary artery bypass surgery in 30 centers. Patients were randomized to rIPC (4 × 5 mininflations and deflations of a blood pressure cuff on the upper arm) or a sham preconditioning procedure. The primary end point was a composite of death from cardiovascular causes, nonfatal myocardial infarction, coronary revascularization or stroke, at 12 months. Remote IPC did not reduce the primary outcome (hazard ratio [HR]: 0.95; 95% CI: 0.79–1.15) or any of the secondary outcomes. Indeed, rIPC did not reduce perioperative myocardial injury, suggesting alternative approaches to modulate mitochondrial membrane permeabilization are necessary in the setting of cardiac surgery.

The effects of rIPC have been examined in two modest sized clinical trials of patients with ST-segment elevation myocardial infarction (STEMI). Bøtker *et al*. [23] randomized 333 STEMI patients to four cycles of upper arm rIPC prior to PCI or PCI alone, with investigator blinding only, observing a small but significant improvement in myocardial salvage index in addition to left ventricular ejection fraction in patients treated with rIPC (47% rIPC cohort vs 43% control), which was most evident in patients with anterior myocardial infarction. Although these changes were no longer evident at 30 days, all-cause mortality and the composite end point of death, myocardial infarction, readmission for heart failure and ischemic stroke/transient ischemic attack were lower in the rIPC group compared with control (HR: 0.32; 95% CI: 0.12–0.88; p = 0.027 and HR: 0.49; 95% CI: 0.27–0.89; p = 0.018, respectively) after 3.8 years of follow-up. Using a similar design, White *et al*. [24] randomized 197 patients to either four cycles of upper arm rIPC prior to coronary angioplasty or sham, observing a 27% reduction in infarct size using cardiac MRI with rIPC. Although promising, these results require to be confirmed in a larger clinical trial before rIPC can be recommended as mainstream treatment for STEMI. To address this, two large multicenter collaborative trials of CONDI-2/EPIC-PPCI (NCT01857414, NCT02342522) are ongoing with results anticipated in 2017 [6]. Additionally, the RIC-STEMI trial (NCT02313961) [6] will add to the existing data, aiming for enrollment of over 400 and randomizing to rIPC or control, with end points of left ventricular ejection fraction and infarct size as well as MACCE at 12 months.

#### Ischemic postconditioning

Ischemic postconditioning, or slow reperfusion, was first demonstrated by Zhao *et al*. in 2003 utilizing a canine artery ligation model, where administration of three cycles of 30 s of reperfusion and reocclusion immediately following prolonged coronary artery occlusion was found to be as effective as ischemic preconditioning at attenuating myocardial reperfusion injury [25]. The therapeutic window for administration of the postconditioning stimulus following the ischemic stimulus appears narrow [26] and, as with IPC, the mPTP appears to be common to both mechanisms of action [27].

Attempts to translate the experimental benefits of postconditioning into the clinical arena have yielded mixed results. An early prospective multicenter trial randomized patients with acute STEMI to primary PCI alone versus primary PCI followed by intermittent coronary artery occlusions (postconditioning) [26]. The authors observed a 36% reduction in infarct size at 72 h by biomarker assay and improvement of blush grade (p < 0.05), although there were no significant improvements in ECG parameters. While these findings have been repeated in other studies [28–32], a similar number have failed to demonstrate a reduction in infarct size [33–36] with one study reporting reduced myocardial salvage with ischemic postconditioning [37]. The meta-analysis by Abdelnoor *et al*. found no net cardioprotective effect from postconditioning, with small sample sizes biasing outcomes of left ventricular ejection fraction [38]. Methodological bias in the form of absence of adequate sham and blinding is present in a minority of the studies but almost all studies contribute bias through small sample size and patient heterogeneity. Larger trial data are awaited from the ongoing DANAMI 3 trial [39].

### • Pharmacological conditioning

A multitude of pharmacological approaches have been developed to reduce the adverse impact of reperfusion injury. A drug which can be administered intravenously to effectively confer postischemic conditioning offers an attractively practical therapeutic option in comparison to invasive ischemic preconditioning and biological approaches. Preclinical data have highlighted the importance of oxidative stress and chemokine expression as potential therapeutic targets for pharmacological agents in STEMI [40]. We present an overview of the relevant agents in recent or ongoing clinical trials.

#### Cyclosporine A

The immunosuppressant drug cyclosporine is well established in clinical use in many inflammatory conditions and post-transplantation. Following on from experimental data demonstrating that cyclosporine A inhibits opening of the mPTP, a small randomized pilot study (n = 58) examined the benefits of cyclosporine A in patients with STEMI [41]. Treatment with cyclosporine A was associated with a 40% reduction in serum creatine kinase, but not troponin, at 5 days and in a subgroup (n = 27), a 20% reduction in infarct size at 6 months. In contrast, the recently published, multicenter, double-blinded, randomized CIRCUS trial enrolled almost 800 patients with STEMI and observed no significant difference in left ventricular ejection fraction, adverse remodeling or clinical outcome at 12 months with intravenous cyclosporine A administered prior to PCI, compared with placebo [42]. Similarly, in the recently published multicenter CYCLE trial [43] (n = 410), pretreatment with cyclosporine A had no effect on ST-segment resolution at 60 min, cardiac biomarker release, left ventricular remodeling or clinical events out to 6 months. A third smaller randomized controlled trial, CAPRI [44], assessing the effect of cyclosporine A in patients with STEMI undergoing PCI will be reported in 2018.

#### Exenatide

Exenatide is a glucagon-like peptide-1 analog used in the treatment of diabetes mellitus [45]. In addition to its hypoglycemic properties, it is thought to have potential benefits in reducing infarct size in myocardial reperfusion injury [46–48]. In a study by Lønborg *et al*. [49], infusion of exenatide initiated prior to primary PCI for STEMI was associated with a 30% relative reduction in infarct size where PCI was performed within 132 min from presentation. The smaller EXAMI study [50] followed this but examined a higher dose of exenatide. It established safety and efficacy at higher dose, but found no change in infarct size or clinical end points. In a further randomized, placebo controlled trial (n = 58) of patients with STEMI, subcutaneous exenatide administered at the time of primary PCI was associated with a significant reduction in infarct size as assessed by biomarker release and cardiac MRI [51]. A Phase II study of exenatide in patients with STEMI, the EMPRES trial (NCT01938235 [6]), is ongoing.

#### Adenosine

Adenosine has been shown to induce cardioprotective effects in the setting of myocardial ischemia through a variety of proposed mechanisms including regulation of heart rate, blood pressure and vasodilatation [52]. The AMISTAD [53] pilot trial randomized 237 patients presenting within 6 h of onset of STEMI to receive a systemic intravenous infusion of either adenosine or placebo, in addition to reperfusion therapy. Compared with placebo, there was a 33% relative reduction in infarct size with adenosine and this benefit was greatest in patients with anterior infarction. The larger Phase III AMISTAD II [54] trial randomized 2118 patients to one of two dosing regimens of adenosine or placebo. Despite a reduction in infarct size with the higher dose of adenosine, this failed to translate into a reduction in clinical end points, including the composite primary end point of new onset congestive heart failure or death within 6 months. More recently, trials of intracoronary adenosine before and after stent deployment have failed to demonstrate any improvement in myocardial salvage index [55] or perfusion with adenosine [56]. The multicenter REFLO-STEMI [57] trial randomized patients with STEMI presenting within 6 h to undergo PCI with either adjunctive intracoronary adenosine or intracoronary sodium nitroprusside or PCI alone (n = 247) and found no difference in infarct size, microvascular obstruction or major adverse cardiovascular events at 6 months between treatment groups.

#### Nitric oxide signaling

Preclinical and early clinical data have demonstrated that nitric oxide has cardioprotective potential by reducing myocardial oxygen demand, increasing coronary blood flow and reducing spasm [58]. The NOMI [59] study randomized 250 patients with acute STEMI to inhaled nitric oxide or placebo. While there was no effect on infarct size, there was a trend to improved recovery of left ventricular function with nitric oxide which became significant at 4 months. Using a different donor, sodium nitroprusside, the multicenter REFLO-STEMI [57] trial failed to demonstrate any benefit compared with placebo in patients with STEMI undergoing primary PCI. The results of a second randomized placebo-controlled Phase II trial of sodium nitroprusside in STEMI, NITRITE-AMI [60], are awaited.

#### Modulating mitochondrial function

As the importance of molecular signaling at the mitochondrial membrane in ischemia reperfusion injury has become clearer, research has been directed toward directly regulating mitochondrial function. A number of promising candidate drugs targeting mitochondrial function have emerged from preclinical work to undergo Phase I and II clinical trials. Protein kinase C isoenzyme, widely considered essential in the mechanism of reperfusion preconditioning, was investigated in the PROTECTION-AMI [61] study, which enrolled 1176 patients with STEMI and randomized them to one of three doses of delcasertib (a specific protein kinase C inhibitor) or placebo. Despite the encouraging experimental data, no effects on extent of myocardial injury or clinical end points were observed with delcasertib. A compound that delays mPTP opening, TRO40303, reduced myocardial infarct size in preclinical models but has failed to live up to expectations in early clinical trials. The randomized controlled MITOCARE [62] study evaluated the efficacy of intracoronary TRO40303 in 163 STEMI patients and found no effect on infarct size or left ventricular function at 1 month. Similarly, MTP-131 (bendavia), a compound that both scavenges reactive oxygen species and inhibits opening of the mPTP, was evaluated in the Phase II EMBRACE-STEMI [63] trial. Enrolling 297 patients with STEMI, EMBRACE-STEMI reported no significant change in infarct size or left ventricular function with administration of MTP-131. Failure to translate encouraging preclinical data to clinical trials with these novel agents has been suggested to relate, at least in part, to unfamiliarity with appropriate dose regimens and lack of sufficient statistical power to clarify trends in clinical end points [62,64].

#### Atrial natriuretic peptide

Atrial natriuretic peptide is a powerful endogenous vasodilator. Kitakaze *et al*. randomized 569 patients with STEMI undergoing primary PCI to either atrial natriuretic peptide or placebo administered as an infusion over 3 days and reported a 14.7% reduction in infarct size by serum biomarker and an increase in left ventricular ejection fraction at 2–8 weeks postinfarct that was still present at 6–12 months [65]. However, atrial natriuretic peptide has been used widely in Japan for treatment of acute heart failure [66] and, in a recent retrospective analysis, a significantly increased in-hospital mortality was identified in patients treated with the drug compared with those who were not [67]. It has also been associated with increased requirement for renal replacement therapy in patients undergoing cardiovascular surgery [68]. These findings mitigate the initial promise of atrial natriuretic peptide in this context and call for larger studies to better characterize its risk–benefit profile in acute myocardial infarction.

#### Other novel agents

The SOLSTICE [69] study investigated losmapimod, a novel inhibitor of the stress-activated kinase p38 MAPK. Despite no significant change in serum biomarkers of infarct size, there was an increase in left ventricular ejection fraction and decrease in end systolic and diastolic pressures both at 3–5 days and 12 weeks postinfarct in a proportion of patients undergoing cardiac MRI. Following this, the ongoing multicenter LATITUDE-TIMI 60 [70] trial aims to enroll >25,000 patients to assess the efficacy and safety of p38 MAPK inhibition with losmapimod in both STEMI and non-STEMI with results expected in December 2018.

The function of the myocardial sodium–hydrogen exchanger in intracellular acid-base regulation has also been implicated in reperfusion injury. Disappointingly, following promise in experimental models, the sodium–hydrogen exchanger inhibitors eniporide and cariporide failed to demonstrate any cardioprotective results although there were no concerning adverse effects [71,72]. More recently, a novel sodium–hydrogen exchanger inhibitor (TY-51924) was trialed in patients with STEMI. While there was no overall improvement in myocardial salvage index with TY-51924, in a *post hoc* analysis, subjects with a large area of myocardium at risk and no anterograde flow did appear to gain some benefit [73]. There is scope for larger studies to clarify the role of sodium–hydrogen exchanger inhibition in cardioprotection.

The compound, FX06, an endogenous peptide derived from human fibrin, has been investigated in the treatment of STEMI on account of its anti-inflammatory properties [58]. The proof-of-concept FIRE [74] study established no effect on infarct size assessed by late gadolinium enhancement at 5 days postinfarction, although a reduction in the size of necrotic core zone on cardiac MRI was observed with FX06.

Bioabsorbable alginate IK-5001 is a polysaccharide polymer produced from brown seaweed that, in the presence of excess ionized calcium present in infarcted myocardium, assembles to form a flexible gel structurally resembling extracellular matrix [75]. In theory, this may provide temporary support to the infarct zone and in an experimental model, reverse left ventricular remodeling postinfarct. Intracoronary IK-5001 injection is currently undergoing investigation in patients with STEMI in the PRESERVATION 1 study (NCT01226563 [6]). Publication of the data is expected in 2020 but preliminary data presented at the European Society of Cardiology Congress 2015 have not indicated any positive effect on adverse left ventricular remodeling compared with placebo at 6 months [76].

### • Metabolic & mechanical strategies

#### Therapeutic hypothermia

Early clinical work has indicated that induction of early mild therapeutic hypothermia may be associated with a reduction in infarct size in patients with acute STEMI [77,78]. The early establishment of hypothermia would seem to be a key determinant of the degree of cardioprotection, with maximum protection in animal models occurring when hypothermia was established prior to ischemic stimulus [79]. Various methods of inducing hypothermia have been explored, with the need to find a balance between rapid induction of hypothermia and an acceptable level of invasiveness to be clinically useful in the context of concurrent reperfusion therapies. The COOL-MI and ICE-IT trials evaluated the combination of endovascular cooling with intravenous cold saline prior to reperfusion therapy in patients with STEMI and reported no overall reduction in infarct size at 30 days [80]. However, in a *post hoc* analysis, there was a suggestion of benefit in patients with anterior myocardial infarction [81]. One of the major concerns with this strategy has been the delay to reperfusion as a result of the cooling process. However, utilizing the same methods, the pilot study RAPID MI-ICE [82] randomized 20 acute myocardial infarction patients to 3 h of therapeutic hypothermia or control, successfully achieving hypothermia (<35°C) prior to reperfusion in 100% of patients (n = 18) with no delay to reperfusion. In this pilot study, cooling was associated with a 37% reduction in infarct size. The larger CHILL-MI [83] study followed this with a shorter period of hypothermia (1 h) and randomized 120 patients with STEMI to hypothermia by either rapid cold saline infusion and endovascular cooling or standard care. The investigators reported no significant reduction in infarct size, but did observe lower incidence of heart failure at 45 days in anterior STEMIs (3% hypothermia vs 14% control), with potential inference of a positive effect in this group. In contrast to endovascular cooling, the multicenter VELOCITY [84] trial used automated peritoneal lavage to induce therapeutic hypothermia, with disappointing results. Fifty-four patients were randomized to 3 h of peritoneal hypothermia or control. No improvement in infarct size was demonstrated, with an increase in both major adverse cardiovascular events and stent thrombosis in the treatment group.

#### Hyperoxemia

The preclinical finding that hyperbaric oxygen delivery can reduce myocardial ischemia-reperfusion injury led to its clinical investigation in AMIHOT I [85]. Hyperoxemia was induced and maintained with an extracorporeal aqueous oxygen circuit. Again, while no significant impact on size of infarct was seen, there was a suggestion of benefit in the anterior STEMI subgroup. AMIHOT II [86] followed this, using Bayesian hierarchical modeling to include some of the AMIHOT I data, and reported a reduction in infarct size by 6.5%, more marked at 10% in the subgroup with left ventricular ejection fraction <40%. The international multicenter DETO2X-AMI trial ([6], NCT01787110), randomizing 6600 patients with STEMI to inhaled oxygen therapy or room air for 6–12 h postinfarction, aims to clarify the role of hyperoxemia in cardioprotection. This follows on from the AVOID trial [87], a multicenter prospective randomized controlled trial of 638 patients with STEMI, which reported a possible deleterious effect of inhaled oxygen in the form of increased early myocardial injury when administered in the absence of hypoxia.

#### Coronary sinus occlusion

Pressure-controlled intermittent coronary sinus occlusion (PICSO) aims to improve myocardial microvascular perfusion following PCI in patients with anterior STEMI. In theory, by occluding the coronary sinus, arterial wedge pressure will rise, increasing perfusion pressure in the infarcted territory. Evaluated in a small safety and feasibility study of 30 patients with STEMI, PICSO had no effect on myocardial recovery or infarct size evaluated by cardiac MRI when compared with matched historical controls [88]. Although there were no major safety issues, logistical difficulties permitted only 12 of 30 patients to undergo the specified 90 min of therapy. When the analysis was restricted to those patients who actually received the treatment, infarct size reduction from 2–5 days to 4 months was greater for patients successfully treated with PICSO compared with matched controls (41.6 ± 8.2% vs 27.7 ± 9.9%, respectively; p = 0.04). Further work is required to evaluate the feasibility and efficacy of PICSO in acute myocardial infarction before any meaningful conclusions can be reached.

## Regenerative therapies

### • Biological therapies

Regenerative medicine has generated substantial interest in recent years. The prospect of developing novel treatments for acute myocardial infarction that stimulate angiogenesis, promote myocardial regeneration and prevent left ventricular dysfunction is highly attractive. Multiple strategies have been proposed ranging from the mobilization of endogenous progenitor cells through the administration of targeted growth factors to engraftment of autologous or allogeneic cells [89]. The structural and functional complexity of myocardium means that simply transplanting stem cells into the infarct zone is unlikely to work in isolation, integration of engrafted tissue with existing myocardial tissue being critical to the restoration of functional myocardium [89]. However, in the face of an incomplete understanding of the complex mechanisms governing stem cell and growth factor regulation and interaction, there has been little consensus on the optimal cell type and route of administration and research approaches have varied hugely. Here, we focus on cell therapies and growth factors that have been evaluated in clinical trials.

#### Cell therapies

The regenerative potential of multiple cell types and sources is being evaluated. These range from pluripotent stem-cell populations capable of differentiation to multiple cardiac cell types, often derived from embryonic stem cells or induced pluripotent stem cells, to adult stem cells including cardiac progenitor cells (CPC), adipose-derived stem cells and bone marrow-derived cells (BMCs). These cells differ widely in their origin, proliferative potential and degree of maturity. The safety and effectiveness of some of these cell therapies have been studied in clinical trials while others await translation to clinical research [90].

Early studies have assessed the effectiveness of adult stem cells (BMC and adipose-derived cells) that are more readily accessible, but less likely to contribute directly to myocardial regeneration. Use of autologous cells avoids the need for immune suppression and circumvents ethical concerns, but these adult stem cells are lineage-committed and less able to differentiate into cardiac cells compared with pluripotent stem cells that can form functional cardiomyocytes, smooth muscle cells and endothelial cells [91]. While CPC populations have been isolated from the adult heart during cardiac surgery, and these cells may be more likely than BMC to regenerate the myocardium, therapeutic use will require cell banking and transplantation of allogeneic cells in to patients with acute myocardial infarction.

#### Bone marrow cells as a source of endothelial progenitor cells

Over a decade ago, Asahara *et al*. defined a putative endothelial progenitor cell, isolated from blood mononuclear cells and thought to arise from the bone marrow, with the potential to home to sites of vascular injury and directly contribute to vascular repair and neovascularization [92–94]. BMC therapies have been evaluated in patients with acute myocardial infarction primarily as they are thought to contain endothelial progenitor cells. Indeed both populations contain cells expressing endothelial surface markers and give risk to cells with an endothelial morphology when cultured under angiogenic conditions [95]. Our understanding of endothelial progenitor cell biology has evolved since these original descriptions and two different populations have been isolated and characterized. Early outgrowth cells or colony forming unit-endothelial progenitor cells are hematopoietic in origin, express leukocyte markers, but do not undergo prolonged proliferation in culture [95]. Despite evidence of indirect paracrine proangiogenic properties, early outgrowth cells do not directly contribute to vascular repair and are no longer considered progeny of a circulating endothelial progenitor cell. In contrast, endothelial outgrowth cells have a typical endothelial morphology and phenotype, lack expression of hematopoietic and leukocyte antigens, can proliferate in culture for prolonged periods and from perfusing vessels *in vivo*, but uncertainty remains as to the origin of their progeny [95].

A myriad of small Phase I and II clinical trials of cell-based therapies in patients with STEMI, with differences in the cell types used and the mode of delivery, have yielded mixed results. Much of the clinical work to date in cell therapies for myocardial infarction has focused on the intracoronary delivery of BMC therapies, but despite a large amount of endeavor, the promise of regenerative cell-based therapies in the setting of acute myocardial infarction has yet to be realized. Meta-analyses of BMC trials performed up to 2008 consistently confirmed a small but statistically significant increase in left ventricular ejection fraction (3.66%; 95% CI: 1.93–5.4%, largest analysis [96]) of cell therapy delivered 7 days postinfarction [97,98]. Of the trials reporting beneficial effects on left ventricular ejection fraction, most enrolled <50 patients to treatment [99,100] and one did not include a sham control and therefore was not double blind [101]. The REPAIR-AMI trial is the largest study to date (n = 204) and, employing a double-blinded sham-controlled design [102] demonstrated a trend toward improvement in left ventricular ejection fraction with cell therapy as assessed by cardiac MRI. The treatment effect was marginal but statistically significant after an adjustment for differences in the left ventricular ejection fraction at baseline assessed by left ventricular angiography. Subsequent studies using cardiac MRI as the sole method for defining the primary outcome failed to demonstrate an effect of cell therapy on left ventricular ejection fraction [103–106]. A random effects meta-analysis of 22 randomized controlled trials of intracoronary BMC therapy following acute myocardial infarction published between 2002 and 2013 [107] observed a small but significant increase in left ventricular ejection fraction of 2.1% compared with control and an associated reduction in infarct size. However, this benefit was lost when the meta-analysis was restricted to studies using cardiac MRI to define left ventricular ejection fraction. Cell therapy was not associated with any effect on MACCE at 6 months.

**Figure 2.**
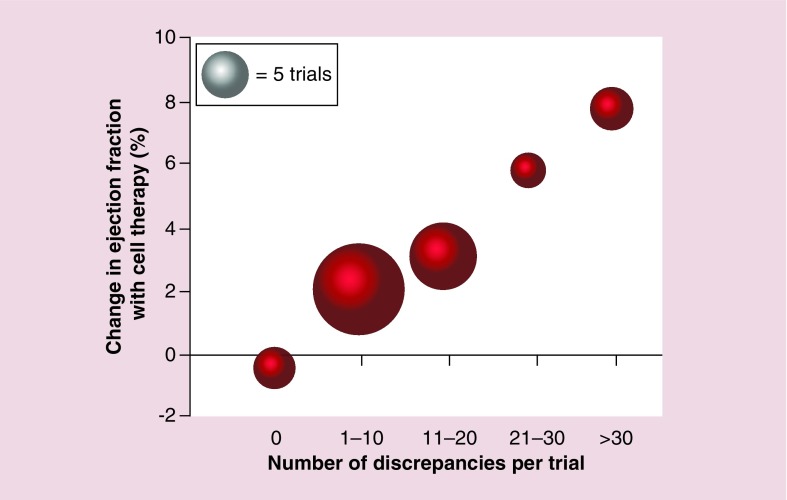
**Mean ejection fraction effect size by number of discrepancies in autologous bone marrow stem cell trials’ reports.** Adapted with permission from [108] © BMJ Publishing Group Limited (2014).

In an effort to explain the marked heterogeneity in reported outcomes with autologous BMC therapies in patients with acute myocardial infarction, the DAMASCENE [108] investigators undertook a weighted-regression meta-analysis of 49 trials, looking for discrepancies in study design, execution, analysis and interpretation. A positive correlation was observed between the number of discrepancies and the reported augmentation in left ventricular ejection fraction with the greatest effect on left ventricular ejection fraction seen in trials with no sham control procedure (Figure 2). Perhaps most notably, the five trials with no discrepancies reported no effect on left ventricular ejection fraction from BMC therapies. It seems likely that the lack of benefit of these therapies is in part explained by the absence of true endothelial progenitor cells in bone marrow. It may be that other cell populations may enhance cardiac function or promote angiogenesis through other mechanisms. It is critical that we understand these mechanisms before embarking on further clinical trials of undifferentiated or mixed cell populations.

#### Mesenchymal stem cells

Mesenchymal stem cells, initially described in bone marrow, have wide differentiation potential and broad tissue distribution [109]. Phase I and II studies have established safety and feasibility [110,111], observing a tendency to improvement in left ventricular ejection fraction and reduction in ventricular dysrhythmia. Ixmyelocel-T is a cell therapy developed from autologous bone marrow where the final expanded product is enriched for macrophages and CD90^+^ mesenchymal stem cells [112]. In a recent multicenter, double-blinded, placebo-controlled trial, 126 patients with ischemic-dilated cardiomyopathy were randomized to receive transcatheter intra-myocardial Ixmyelocel-T or placebo, with a composite primary end point of all-cause mortality, cardiovascular admissions or attendances for heart failure. While the investigators reported a significant reduction in the primary end point in patients receiving Ixmyelocel-T, surprisingly this was not associated with improvements in left ventricular volume or ejection fraction.

#### Cardiac progenitor cells

CPCs are resident in adult cardiac tissue and have been evaluated in patients with ischemic cardiomyopathy. There is no consensus as to the optimal method to define these cells, but the surface expression of c-kit (stem-cell factor) provides an opportunity to isolate putative CPC populations from the human heart. The SCIPIO trial [113] enrolled 33 patients who underwent isolation and expansion of c-kit+ cells from the right atrial appendage harvested during coronary artery bypass surgery. The expanded cell product was subsequently infused into the vessel supplying the ischemic territory. The investigators reported an improved global and regional left ventricular ejection fraction, increase in viable left ventricular mass by cardiac MRI and a reduction in scar size at 4 months, persisting out to 1 year. However, enthusiasm for these encouraging preliminary findings has been diminished by questions raised after publication that have resulted in an expression of concern and retraction of associated work [114,115]. More recently, the CADUCEUS trial [116] evaluated the feasibility and safety of cardiosphere-derived cells in acute myocardial infarction, randomizing 31 patients to intracoronary cell therapy or routine care. Technical difficulties and patient withdrawals resulted in only 17 patients being randomized to treatment and eight patients to control. While left ventricular ejection fraction was unchanged at 1 year, the investigators observed a trend toward smaller scar size and an increase in viable myocardium in patients receiving cell therapy.

#### Optimal administration parameters

As is the case with the type and timing of administration of stem-cell products, optimal dosing and route of administration have yet to be established and this may be partly responsible for the inconsistency of the outcomes to date. The pharmacokinetics and dynamics of live cell-based therapies are fluid and complex so have yet to become sufficiently well understood to provide optimal dosing and route of administration; there have been no studies to date that have simultaneously examined both dose and route [117]. The two most extensively investigated routes of administration have been endovascular (usually intracoronary) and intramyocardial (either surgically or transcatheter) with a tendency to see more encouraging results from the former route. Advances in technological approaches to cell harvest and administration coupled with evolving understanding of the complex biological interplay of stem-cell dynamics lend promise to the development of innovative cell therapy approaches in the future.

#### Growth factors & cytokines

The role of cytokine-mediated tissue growth at the peri-infarct zone and stimulation of endogenous myocardium in regulation of the response to injury is increasingly well recognized [118]. It is becoming increasingly apparent that paracrine actions of engrafted cells combined with growth factor-mediated signaling are likely to play a key role in the success of any biological therapy. Indeed, growth factors have received attention in their own right as potential therapeutic targets for myocardial regeneration following infarction [119]. While a detailed review of cardiac growth factors can be found elsewhere [120], there have been a small number of clinical trials in patients with acute myocardial infarction. Exogenous recombinant erythropoietin has been studied as a growth factor therapy in myocardial infarction and initially encouraging animal data lead to a series of clinical studies, although these failed to demonstrate any benefit [121]. Talan *et al*. explored the reasons for this failure in an animal model designed to specifically assess impact of the drug when given at varying time intervals and would more accurately reflect those of the clinical studies [121]. They found a reduction in infarct size of >50% in animals where erythropoietin was administered at the time of reperfusion (2-h duration) but no effect on infarct size was seen with administration at 4 h or greater after reperfusion. This supports the theory that the therapeutic window for some therapies may be very narrow; a variable that further research in this field would need to recognize. In addition, animal models are well known to be limited in lacking the substrate for disease seen in humans, with absence of comorbidity and comparable pathophysiology. Other suggested contributing factors to the failure to translate to clinical research are insufficient sample size, lacking animal data on dose response, patient heterogeneity, variable routes of administration and forms of erythropoietin as well as potentially confounding concurrent treatments [122]. To date, research into the role of growth factors has been largely preclinical and strategies utilizing growth factors alone are few. With further knowledge of the role of growth factors in myocardial injury, future options may explore their potential when co-administered with stem-cell therapies.

#### Challenges facing biological therapies in acute myocardial infarction

Following a large number of studies in the field over the past two decades, the initial enthusiasm in biological therapies is moderated by the complexity of the molecular processes regulating cellular repair and regeneration. In general, there have been few safety concerns raised by the clinical studies carried out to date. Although tumor formation and ectopic tissue growth were encountered in animal studies [123], this has not been a major safety issue in clinical studies. Methodological and technical complications may account for much of the heterogeneity observed in the trials to date, as may small sample sizes. Larger, rigorously conducted and more specific trial data are needed to further improve our understanding of the optimal type of stem cell, dosage and route of administration as well as allay medium and longer term safety concerns. In addition, there is evidence that differences in intermolecular signaling pathways associated with advancing age may alter the pharmacokinetics and dynamics further indicating that patient selection is of key importance [124]. In summary, evidence of a robust, clinically significant improvement in left ventricular systolic function following stem-cell therapy in patients with acute myocardial infarction is limited.

## Conclusion & future perspective

Encouraging preclinical data has often garnered great enthusiasm for development of novel therapies in myocardial infarction, but translating these data in to the clinical arena has often proven challenging. The limitation of animal models of myocardial injury with the absence of substrate for cardiovascular disease, plaque rupture, thrombosis and microembolization is well established, and many clinical studies fail to reach statistical power in recruitment with diverse patient demographics and myocardial injury profiles. The use of cardiac MRI as the gold standard of quantifying change in left ventricular ejection fraction is increasingly accepted, although it is conceivable that the more relevant end point is long-term clinical outcomes.

Understanding the physiology of cell signaling in myocardial repair continues to evolve with the potential to progress some biological regenerative therapies. Most cells in the body secrete small membrane-bound vesicles, exosomes, which can regulate intercellular microcommunication in both normal and pathological states [125]. Exosomes and microvesicles derived from stem cells contain various types of miRNAs, growth factors and cell-protection components believed to be fundamental to the cardioprotective potential of these cell therapies [125,126], suppressing apoptosis in the infarct zone as well as promoting neovascularization and chemoattraction of cardiac progenitor cells [127,128]. These exosome-secreted factors are cell-free and may offer cardioprotective and regenerative potential without the need to harvest, expand and deliver stem cells to the heart. Ongoing research investigates mRNAs as biomarkers for acute myocardial infarction (NCT02751060), looks to clarify their role in efficacy of antiplatelet therapy (NCT02071966) and aims to better understand the role of mRNAs and microvesicles in acute myocardial infarction and left ventricular remodeling (NCT01875484). Greater understanding in these areas may then lead to development of therapeutic strategies utilizing mRNAs and microvesicles for myocardial infarction.

Treatment with oral P2Y12 inhibitors is central to the current treatment of patients with acute myocardial infarction. While the benefit of P2Y12 inhibition has been largely attributed to their antiplatelet effect, there are emerging preclinical data to suggest a cardioprotective role in the setting of ischemia-reperfusion injury [129,130]. Given the importance of timing in the delivery of treatments for acute myocardial infarction, rapid P2Y12 inhibition with intravenous administration of cangrelor, thus avoiding delays from gut absorption or metabolic conversion, may afford an opportunity to maximize the cardioprotective potential of P2Y12 inhibition while retaining the antiplatelet benefits [130]. Future work may confirm these benefits in clinical practice. Cangrelor has been trialed in the PCI setting yielding mixed results for clinical end points. As yet there has been no trial of cangrelor in STEMI with primary end point of myocardial salvage [131].

Major progress has been made in the development and testing of a wide range of therapies to reduce the impact of reperfusion injury in acute myocardial infarction (summarized in Table 1). Results of key trials are awaited with the hope of providing clarity with greater sample sizes and robust study design (summarized in Table 2). These trials and other novel approaches offer hope to the next generation of clinical therapies in the battle against reperfusion injury.

**Table 1.** **Key trials and meta-analyses of novel therapeutic strategies in myocardial infarction.**

**Novel therapy**	**Study (year)/meta-analysis**	**Number enrolled, study design**	**Intervention for STEMI**	**End points**	**Outcomes/notes**	**Ref.**
***Cardioprotective therapies***						

rIPC	Bøtker *et al*. (2010)	333, single-center RCT	rIPC (4 × 5 min cycles) vs standard care	MSI at 30 days	Small significant increase in MSI and EF	[23]
	White *et al*. (2015)	197	rIPC (4 × 5 min cycles) vs sham pre-PPCI	MI size by CMR at 3–6 days	Reduction in MI size in rIPC arm	[24]
	Hausenloy DJ *et al*. (2015) ERICCA	1612, multicenter RCT	rIPC (4 × 5 min cycles) vs sham pre-PPCI	Cardiovascular death, nonfatal MI, PCI/CABG or stroke (1 year)	No significant difference in primary end point	[22]

IPost	Abdelnoor *et al*. (2014)	21, RCTs random-effects meta-analysis	Various regimes of CA occlusion	MI size by CMR, serum biomarkers, ECG changes	No significant cardioprotection Possible benefit on HF incidence	[38]

Cyclosporine A	Cung TT *et al*. CIRCUS	791, multicenter, double-blinded RCT	iv. cyclosporine pre-PPCI vs placebo	Death, worsening HF or admission, adverse LV remodeling	No significant difference in primary end point	[42]
	Ottani F *et al*. (2016) CYCLE	410, multicenter RCT	iv. cyclosporine pre-PPCI vs standard care	≥70% ST resolution (60 min), serum biomarkers, LV remodeling, clinical end points to 6 months	No significant difference in any end point	[43]

Exenatide	Woo JS *et al*. (2013)	58, placebo-controlled RCT	SC exenatide at PPCI vs placebo	MI size and LV function by CMR (1 month), serum biomarkers (3–6 days)	Significant improvement in MI size and EF 18 patients in treatment arm	[51]

Adenosine	Ross AM *et al*. (2005) AMISTAD-II	2118, double-blind, placebo-controlled RCT	iv. adenosine (two dose regimes) vs placebo	New HF or hospitalization at 6 months	No reduction in primary end point despite reduced MI size at higher dose	[54]

Adenosine/nitroprusside	Nazir SA *et al*. (2015) REFLO-STEMI	247, multicenter RCT	IC adenosine or SNP at PPCI vs standard care	MI size by CMR	No change on MI size observed with either treatment	[57]

Hypothermia	Erlinge D *et al*. (2014) CHILL-MI	120, multicenter RCT	Endovascular cooling vs standard care	MI size by CMR at 4 days	No change in MI size observed	[83]

Hyperoxemia	Stone GW *et al*. (2009) AMIHOT-II	301, multicenter RCT	90 min IC SSO_2_ to LAD vs standard care	MI size by SPECT	Significantly reduced MI size More marked in LVEF <40% Noninferior rates of MACE	[86]

***Regenerative therapies***						

BMC therapy	De Jong R *et al*. (2015)	22, trials random-effects meta-analysis	BMC therapy in acute MI	LVEF, LV volumes, MI size, MACE	Small significant reduction in MI size, with increased EF Benefit lost when restricted to CMR studies	[107]
	Nowbar AN *et al*. (2014) DAMASCENE	49, trials meta-analysis	BMC therapy in acute MI	Trial discrepancies correlation with reported effect on effect size	Strong correlation between number of discrepancies and reported improvement in LVEF	[108]

BMC: Bone marrow cell; CA: Coronary artery; CABG: Coronary artery bypass grafting; CMR: Cardiac magnetic resonance; EF: Ejection fraction; HF: Heart failure; IC: Intracoronary; IPost: Ischemic postconditioning; iv.: Intravenous; LAD: Left anterior descending artery; LV: Left ventricle/ventricular; LVEF: Left ventricular ejection fraction; MACE: Major adverse cardiovascular event; MI: Myocardial infarction; MSI: Myocardial salvage index; PCI: Percutaneous coronary intervention; PPCI: Primary percutaneous coronary intervention; RCT: Randomized controlled trial; rIPC: Remote ischemic preconditioning; SC: Subcutaneous; SNP: Sodium nitroprusside; SPECT: Single-photon emission computed tomography; SSO_2_: Supersaturated oxygen; ST: ST-segment; STEMI: ST-elevation myocardial infarction.

**Table 2.** **Major ongoing clinical trials of novel cardioprotective therapies.**

**Trial**	**Estimated enrollment**	**Design**	**Intervention**	**Primary outcome**	**Estimated final completion**	**Ref.**
RIC-STEMI (NCT02313961)	492	RCT	Lower limb RIPC prior to ≥10 min prior to angioplasty vs no intervention	Cardiac mortality or HF admission at 1 year	May 2017	[132]

CONDI-2/ERIC-PPCI (NCT01857414/NCT02342522)	4300/2000	Single/double-blinded RCT	4 × 5 min cycles limb RIPC prehospital vs no intervention/sham	Cardiac mortality or HF admission at 1 year	December 2017/December 2019	[133,134]

DANAMI-3	2000	Single-blinded RCT	4 × 30 s cycles culprit vessel occlusion prior to stent deployment vs no intervention	All-cause mortality and heart failure	February 2021	[39]

EMPRES (NCT01938235)	198	Double-blinded RCT	iv. exenatide bolus (pre-PPCI) and 24 h infusion (post-PPCI) vs placebo	Infarct size: area at risk ratio (by CMR)	January 2017	[135]

CAPRI (NCT02390674)	68	Double-blinded RCT	Single dose iv. cyclosporine prior to PCI vs placebo	Infarct size at 12 weeks (by CMR)	March 2018	[136]

DETO2X-AMI (NCT01787110)	6650	RCT	Continuous inhaled oxygen (6 l/min) for 6–12 h from inclusion vs no intervention	All-cause mortality at 1 year	September 2017	[137]

NITRITE-AMI	80	Double-blinded RCT	iv. sodium nitrite during PPCI vs placebo	Infarct size (by biomarker AUC) to 48 h	Awaited	[60]

AUC: Area under the curve; CMR: Cardiac magnetic resonance; HF: Heart failure; iv.: Intravenous; PCI: Percutaneous coronary intervention; PPCI: Primary percutaneous coronary intervention; RCT: Randomized controlled trial; RIPC: Remote ischemic preconditioning.

**EXECUTIVE SUMMARY**

**Ischemic conditioning**

- Utility of local ischemic conditioning is limited by its invasive nature.
- Remote ischemic preconditioning has shown reduction in infarct size and improved long-term clinical outcomes; large-scale clinical trials are ongoing.
- Ischemic postconditioning has yielded mixed results; large-scale trial is ongoing.

**Pharmacological conditioning**

- Cyclosporine A and exenatide are undergoing clinical trial to clarify early negative clinical studies.
- Promise shown by atrial natriuretic peptide is mitigated by concerns over increased in-hospital mortality.
- A variety of innovative and novel agents are still undergoing clinical trial.

**Mechanical & metabolic strategies**

- Cooling confers no significant improvement in left ventricular function overall, but may benefit anterior ST-segment elevation myocardial infarctions and reduce incidence of heart failure.
- Ongoing clinical trial aims to clarify potential benefit from hyperoxemia.
- The role of coronary sinus devices has yet to be established.

**Biological therapies**

- A wide variety of stem cells have undergone preclinical and clinical trial.
- No consensus is reached on optimal administration and dose regimes.
- Realizing the potential of biological therapies will depend on continued understanding of intermolecular signaling pathways and cellular dynamics.

**Future perspective**

- Significant progress has been made in pursuit of effective therapies against myocardial reperfusion injury.
- Realization of the promise of cellular therapy may be achieved with a better understanding of the cellular mechanisms of myocardial repair.
- Modification of gene expression may offer a more widely applicable alternative strategy for myocardial regeneration.

## References

[1] World Health Organization Global status report on noncommunicable diseases 2014. www.who.int/nmh/publications/ncd-status-report-2014/en/.

[2] (2012). Acute myocardial infarction in patients presenting with ST-segment elevation (management of) ESC Clinical Practice Guidelines. *Eur. Heart J.*.

[3] (2015). Acute coronary syndromes (ACS) in patients presenting without persistent ST-segment elevation (management of) ESC Clinical Practice Guidelines. *Eur. Heart J.*.

[4] (2014). ESC/EACTS guidelines in myocardial revascularisation (Guidelines for) ESC Clinical Practice Guidelines. *Eur. Heart J.*.

[5] (2012). Acute and chronic heart failure ESC Clinical Practice Guidelines. *Eur. Heart J.*.

[6] ClinicalTrials.gov https://www.clinicaltrials.gov/.

[7] Hausenloy DJ, Yellon DM (2016). Ischaemic conditioning and reperfusion injury. *Nat. Rev. Cardiol.*.

[8] Carden DL, Granger DN (2000). Pathophysiology of ischaemia-reperfusion injury. *J. Pathol.*.

[9] Park JL, Lucchesi BR (1999). Mechanisms of myocardial reperfusion injury. *Ann. Thorac. Surg.*.

[10] Kroemer G, Galluzzi L, Brenner C (2007). Mitochondrial membrane permeabilization in cell death. *Physiol. Rev.*.

[11] Cellier L, Tamareille S, Kalakech H (2016). Remote ischemic conditioning influences mitochondrial dynamics. *Shock*.

[12] Murry CE, Jennings RB, Reimer KA (1986). Preconditioning with ischemia: a delay of lethal cell injury in ischemic myocardium. *Circulation*.

[13] Yellon DM, Downey JM (2003). Preconditioning the myocardium: from cellular physiology to clinical cardiology. *Physiol. Rev.*.

[14] Heusch G (2015). Molecular basis of cardioprotection: signal transduction in ischemic pre-, post-, and remote conditioning. *Circ. Res.*.

[15] Hausenloy DJ (2013). Cardioprotection techniques: preconditioning postconditioning and remote conditioning (basic science). *Curr. Pharm. Design.*.

[16] Przyklenk K, Bauer B, Ovize M, Kloner RA, Whittaker P (1993). Regional ischemic “preconditioning” protects remote virgin myocardium from subsequent sustained coronary occlusion. *Circulation*.

[17] Walsh SR, Tang TY, Kullar P, Jenkins DP, Dutka DP, Gaunt ME (2008). Ischaemic preconditioning during cardiac surgery: systematic review and meta-analysis of perioperative outcomes in randomised clinical trials. *Eur. J. Cardiothorac. Surg.*.

[18] Healy DA, Carroll PJ, Moloney MC (2013). Systematic review and meta-analysis of remote ischaemic preconditioning in percutaneous coronary intervention. *IJC Metabolic Endocrine*.

[19] D'Ascenzo F, Moretti C, Omedè P (2014). Cardiac remote ischaemic preconditioning reduces periprocedural myocardial infarction for patients undergoing percutaneous coronary interventions: a meta-analysis of randomised clinical trials. *EuroIntervention*.

[20] Pei H, Wu Y, Wei Y, Yang Y, Teng S, Zhang H (2014). Remote ischemic preconditioning reduces perioperative cardiac and renal events in patients undergoing elective coronary intervention: a meta-analysis of randomized trials. *PLoS ONE*.

[21] Hoole SP, Heck PM, Sharples L (2009). Cardiac Remote Ischemic Preconditioning in Coronary Stenting (CRISP Stent) study: a prospective, randomized control trial. *Circulation*.

[22] Hausenloy DJ, Candilio L, Evans R (2015). Remote ischemic preconditioning and outcomes of cardiac surgery. *N. Eng. J. Med.*.

[23] Bøtker HE, Kharbanda R, Schmidt MR (2010). Remote ischaemic conditioning before hospital admission, as a complement to angioplasty, and effect on myocardial salvage in patients with acute myocardial infarction: a randomised trial. *Lancet*.

[24] White SK, Frohlich GM, Sado DM (2015). Remote ischemic conditioning reduces myocardial infarct size and edema in patients with ST-segment elevation myocardial infarction. *JACC Cardiovasc. Interv.*.

[25] Zhao ZQ, Corvera JS, Halkos ME (2003). Inhibition of myocardial injury by ischemic postconditioning during reperfusion: comparison with ischemic preconditioning. American Journal of Physiology. *Heart Circ. Physiol.*.

[26] Staat P, Rioufol G, Piot C (2005). Postconditioning the human heart. *Circulation*.

[27] Javadov SA, Clarke S, Das M, Griffiths EJ, Lim KHH, Halestrap AP (2003). Ischaemic preconditioning inhibits opening of mitochondrial permeability transition pores in the reperfused rat heart. *J. Physiol.*.

[28] Ma X, Zhang X, Li C, Luo M (2006). Effect of postconditioning on coronary blood flow velocity and endothelial function and LV recovery after myocardial infarction. *J. Interv. Cardiol.*.

[29] Yang XC, Liu Y, Wang LF (2007). Reduction in myocardial infarct size by postconditioning in patients after percutaneous coronary intervention. *J. Invasive Cardiol.*.

[30] Thibault H, Piot C, Staat P (2008). Long-term benefit of postconditioning. *Circulation*.

[31] Lonborg J, Kelbaek H, Vejlstrup N (2010). Cardioprotective effects of ischemic postconditioning in patients treated with primary percutaneous coronary intervention, evaluated by magnetic resonance. *Circ. Cardiovasc. Interv.*.

[32] Araszkiewicz A, Grygier M, Pyda M (2014). Postconditioning reduces enzymatic infarct size and improves microvascular reperfusion in patients with ST-segment elevation myocardial infarction. *Cardiology*.

[33] Sorensson P, Saleh N, Bouvier F (2010). Effect of postconditioning on infarct size in patients with ST elevation myocardial infarction. *Heart*.

[34] Tarantini G, Favaretto E, Marra MP (2012). Postconditioning during coronary angioplasty in acute myocardial infarction: the POST-AMI trial. *Int. J. Cardiol.*.

[35] Eitel I, Stiermaier T, Rommel KP (2015). Cardioprotection by combined intrahospital remote ischaemic perconditioning and postconditioning in ST-elevation myocardial infarction: the randomized LIPSIA CONDITIONING trial. *Eur. Heart J.*.

[36] Dwyer NB, Mikami Y, Hilland D (2013). No cardioprotective benefit of ischemic postconditioning in patients with ST-segment elevation myocardial infarction. *J. Interv. Cardiol.*.

[37] Freixa X, Bellera N, Ortiz-Pérez JT (2012). Ischaemic postconditioning revisited: lack of effects on infarct size following primary percutaneous coronary intervention. *Eur. Heart J.*.

[38] Abdelnoor M, Sandven I, Limalanathan S, Eritsland J (2014). Postconditioning in ST-elevation myocardial infarction: a systematic review, critical appraisal, and meta-analysis of randomized clinical trials. *Vasc. Health Risk Manag.*.

[39] Høfsten DE, Kelbæk H, Helqvist S (2015). The third DANish study of optimal acute treatment of patients with ST-segment elevation myocardial infarction: ischemic postconditioning or deferred stent implantation versus conventional primary angioplasty and complete revascularization versus treatment of culprit lesion only: rationale and design of the DANAMI trial program. *Am. Heart J.*.

[40] Montecucco F, Carbone F, Schindler TH (2015). Pathophysiology of ST-segment elevation myocardial infarction: novel mechanisms and treatments. *Eur. Heart J.*.

[41] Piot C, Croisille P, Staat P (2008). Effect of cyclosporine on reperfusion injury in acute myocardial infarction. *N. Engl. J. Med.*.

[42] Cung TT, Morel O, Cayla G (2015). Cyclosporine before PCI in patients with acute myocardial infarction. *N. Engl. J. Med.*.

[43] Ottani F, Latini R, Staszewsky L (2016). Cyclosporine A in reperfused myocardial infarction. *J. Am. Coll. Cardiol.*.

[44] Ciclosporin to Reduce Reperfusion Injury in Primary PCI (CAPRI). https://www.clinicaltrials.gov/ct2/show/NCT02390674.

[45] Bułdak Ł, Machnik G, Bułdak RJ, Łabuzek K, Bołdys A, Belowski D (2016). Exenatide (a GLP-agonist) expresses anti-inflammatory properties in cultured human monocytes/macrophages in a protein kinase A and B/Akt manner. *Pharmacol. Rep.*.

[46] Bose AK, Mocanu MM, Carr RD, Brand CL, Yellon DM (2005). Glucagon-like peptide can directly protect the heart against ischemia/reperfusion injury. *Diabetes*.

[47] Timmers L, Henriques JP, de Kleijn DP (2009). Exenatide reduces infarct size and improves cardiac function in a porcine model of ischemia and reperfusion injury. *J. Am. Coll. Cardiol.*.

[48] Hausenloy DJ, Whittington HJ, Wynne AM (2013). Dipeptidyl peptidase-inhibitors and GLP-reduce myocardial infarct size in a glucose-dependent manner. *Cardiovasc. Diabetol.*.

[49] Lønborg J, Kelbæk H, Vejlstrup N (2012). Exenatide reduces final infarct size in patients with ST-segment-elevation myocardial infarction and short-duration of ischemia. *Circ. Cardiovasc. Interv.*.

[50] Bernink FJP, Timmers L, Diamant M (2013). Effect of additional treatment with EXenatide in patients with an Acute Myocardial Infarction: The EXAMI study. *Int. J. Cardiol.*.

[51] Woo JS, Kim W, Ha SJ (2013). Cardioprotective effects of exenatide in patients with ST-segment-elevation myocardial infarction undergoing primary percutaneous coronary intervention: results of exenatide myocardial protection in revascularization study. *Arterioscler. Thromb. Vasc. Biol.*.

[52] Kloner RA (2013). Current state of clinical translation of cardioprotective agents for acute myocardial infarction. *Circ. Res.*.

[53] Mahaffey KW, Puma JA, Barbagelata NA (1999). [for the AMISTAD Investigators]. Adenosine as an adjunct to thrombolytic therapy for acute myocardial infarction results of a multicenter, randomized, placebo-controlled trial: The Acute Myocardial Infarction STudy of ADenosine (AMISTAD) trial. *J. Am. Coll. Cardiol.*.

[54] Ross AM, Gibbons RJ, Stone GW, Kloner RA, Alexander RW (2005). A randomized, double-blinded, placebo-controlled multicenter trial of adenosine as an adjunct to reperfusion in the treatment of acute myocardial infarction (AMISTAD-II). *J. Am. Coll. Cardiol.*.

[55] Desmet W, Bogaert J, Dubois C (2011). High-dose intracoronary adenosine for myocardial salvage in patients with acute ST-segment elevation myocardial infarction. *Eur. Heart J.*.

[56] Fokkema ML, Vlaar PJ, Vogelzang M (2009). Effect of high-dose intracoronary adenosine administration during primary percutaneous coronary intervention in acute myocardial infarction: a randomized controlled trial. *Circ. Cardiovasc. Interv.*.

[57] Nazir SA, Khan J, Mahmoud IZ (2015). Adenosine and sodium nitroprusside versus control for the attenuation of infarct size and microvascular obstruction: the REFLO-STEMI trial. *J. Am. Coll. Cardiol.*.

[58] Fordyce CB, Gersh BJ, Stone GW, Granger CB (2015). Novel therapeutics in myocardial infarction: targeting microvascular dysfunction and reperfusion injury. *Trends Pharmacol. Sci.*.

[59] Janssens S (1 September 2014). Effects of nitric oxide for inhalation in myocardial infarction size (NOMI). *European Society of Cardiology Congress*.

[60] Jones DA, Andiapen M, Van-Eijl TJA (2013). The safety and efficacy of intracoronary nitrite infusion during acute myocardial infarction (NITRITE-AMI): study protocol of a randomised controlled trial. *BMJ Open*.

[61] Lincoff AM, Roe M, Aylward P (2014). Inhibition of delta-protein kinase C by delcasertib as an adjunct to primary percutaneous coronary intervention for acute anterior ST-segment elevation myocardial infarction: results of the PROTECTION AMI randomized controlled trial. *Eur. Heart J.*.

[62] Atar D, Arheden H, Berdeaux A (2015). Effect of intravenous TROas an adjunct to primary percutaneous coronary intervention for acute ST-elevation myocardial infarction: MITOCARE study results. *Eur. Heart J.*.

[63] Gibson CM, Giugliano RP, Kloner RA (2015). EMBRACE STEMI study: a Phase 2a trial to evaluate the safety, tolerability, and efficacy of intravenous MTP-on reperfusion injury in patients undergoing primary percutaneous coronary intervention. *Eur. Heart J.*.

[64] Bulluck H, Yellon DM, Hausenloy DJ (2015). Reducing myocardial infarct size: challenges and future opportunities. *Heart*.

[65] Kitakaze M, Asakura M, Kim J (2007). Human atrial natriuretic peptide and nicorandil as adjuncts to reperfusion treatment for acute myocardial infarction (J-WIND): two randomised trials. *Lancet*.

[66] Saito Y (2010). Roles of atrial natriuretic peptide and its therapeutic use. *J. Cardiol.*.

[67] Matsue Y, Kagiyama N, Yoshida K (2015). Carperitide is associated with increased in-hospital mortality in acute heart failure: a propensity score-matched analysis. *J. Card. Fail.*.

[68] Sasabuchi Y, Yasunaga H, Matsui H, Lefor AK, Fushimi K, Sanui M (2015). Carperitide increases the need for renal replacement therapy after cardiovascular surgery. *J. Cardiothorac. Vasc. Anesth.*.

[69] Newby LK, Marber MS, Melloni C (2014). Losmapimod, a novel pmitogen-activated protein kinase inhibitor, in non-ST-segment elevation myocardial infarction: a randomised Phase trial. *Lancet*.

[70] O'Donoghue ML, Glaser R, Aylward PE (2015). Rationale and design of the LosmApimod To Inhibit p38 MAP kinase as a TherapeUtic target and moDify outcomes after an acute coronary syndromE trial. *Am. Heart J.*.

[71] Zeymer U, Suryapranata H, Monassier JP (2001). The Na+/H+ exchange inhibitor eniporide as an adjunct to early reperfusion therapy for acute myocardial infarction. Results of the evaluation of the safety and cardio-protective effects of eniporide in acute myocardial infarction (ESCAMI) trial. *J. Am. Coll. Cardiol.*.

[72] Mentzer RM, Bartels C, Bolli R (2008). EXPEDITION study investigators. Sodium-hydrogen exchange inhibition by cariporide to reduce the risk of ischemic cardiac events in patients undergoing coronary artery bypass grafting: results of the EXPEDITION study. *Ann. Thorac Surg.*.

[73] Kimura K, Nakao K, Shibata Y (2015). Randomized controlled trial of TY-51924, a novel hydrophilic NHE inhibitor, in acute myocardial infarction. *J. Cardiol.*.

[74] Atar D, Petzelbauer P, Schwitter J (2009). Effect of intravenous FX06 as an adjunct to primary percutaneous coronary intervention for acute ST-segment elevation myocardial infarction. *J. Am. Coll. Cardiol.*.

[75] Leor J, Tuvia S, Guetta V (2009). Intracoronary injection of in situ forming alginate hydrogel reverses left ventricular remodeling after myocardial infarction in Swine. *J. Am. Coll. Cardiol.*.

[76] Zeymer U, Rao SV, Krucoff MW (29 August–2 September 2015). PRESERVATION I: bioabsorbable cardiac matrix for the prevention of remodeling of the ventricle after large ST-segment elevation myocardial infarction. *European Society of Cardiology (ESC) Congress*.

[77] Götberg M, Olivecrona GK, Engblom H (2008). Rapid short-duration hypothermia with cold saline and endovascular cooling before reperfusion reduces microvascular obstruction and myocardial infarct size. *BMC Cardiovasc. Disord.*.

[78] Erlinge D, Götberg M, Grines C (2013). A pooled analysis of the effect of endovascular cooling on infarct size in patients with ST-elevation myocardial infarction. *EuroIntervention*.

[79] Kanemoto S, Matsubara M, Noma M (2009). Mild hypothermia to limit myocardial ischemia-reperfusion injury: importance of timing. *Ann. Thorac Surg.*.

[80] Stone GW, Dixon SR, Grines CL (2007). Predictors of infarct size after primary coronary angioplasty in acute myocardial infarction from pooled analysis from four contemporary trials. *Am. J. Cardiol.*.

[81] O'Neill WW (15–19 September 2003). Cooling as an adjunctive therapy to percutaneous intervention in patients with acute myocardial infarction (COOLMI). *Transcatheter Cardiovascular Therapeutics Session*.

[82] Götberg M, Olivecrona GK, Koul S (2010). A pilot study of rapid cooling by cold saline and endovascular cooling before reperfusion in patients with ST-elevation myocardial infarction. *Circ. Cardiovasc. Interv.*.

[83] Erlinge D, Götberg M, Lang I (2014). Rapid endovascular catheter core cooling combined with cold saline as an adjunct to percutaneous coronary intervention for the treatment of acute myocardial infarction. *J. Am. Coll. Cardiol.*.

[84] Nichol G, Strickland W, Shavelle D (2015). Prospective, multicenter, randomized, con- trolled pilot trial of peritoneal hypothermia in patients with ST-segment-elevation myocardial infarction. *Circ. Cardiovasc. Interv.*.

[85] O'Neill WW, Martin JL, Dixon SR (2007). Acute myocardial infarction with hyperoxemic therapy (AMIHOT). *J. Am. Coll. Cardiol.*.

[86] Stone GW, Martin JL, de Boer MJ (2009). Effect of supersaturated oxygen delivery on infarct size after percutaneous coronary intervention in acute myocardial infarction. *Circ. Cardiovasc. Interv.*.

[87] Stub D, Smith K, Bernard S (2015). Air versus oxygen in ST-segment-elevation myocardial infarction. *Circulation*.

[88] van de Hoef TP, Nijveldt R, van der En M (2014). TCT-Pressure-controlled Intermittent Coronary Sinus Occlusion (PICSO) in acute ST-segment elevation myocardial infarction: final results of the prepare RAMSES study. *J. Am. Coll. Cardiol.*.

[89] Satessa GD, Lenjisa JL, Gebremariam ET, Woldu MA (2015). Stem cell therapy for myocardial infarction: challenges and prospects. *J. Stem Cell Res. Ther.*.

[90] Naaijkens BA, van Dijk A, Kamp O (2014). Therapeutic application of adipose derived stem cells in acute myocardial infarction: lessons from animal models. *Stem Cell Rev. Rep.*.

[91] Kim PJ, Yang PC (2012). Bone marrow cell therapy in clinical trials: a review of the literature. *Rev. Recent Clin. Trials*.

[92] Asahara T, Murohara T, Sullivan A (1997). Isolation of putative progenitor endothelial cells for angiogenesis. *Science*.

[93] Asahara T, Kawamoto A (2004). Endothelial progenitor cells for postnatal vasculogenesis. *Am. J. Physiol. Cell Physiol.*.

[94] Padfield GJ, Newby DE, Mills NL (2010). Understanding the role of endothelial progenitor cells in percutaneous coronary intervention. *J. Am. Coll. Cardiol.*.

[95] Tura O, Skinner EM, Barclay GR (2013). Late outgrowth endothelial cells resemble mature endothelial cells and are not derived from bone marrow. *Stem Cells*.

[96] Abdel-Latif A, Bolli R, Tleyjeh IM (2007). Adult bone marrow–derived cells for cardiac repair: a systematic review and meta-analysis. *Arch. Intern. Med.*.

[97] Martin-Rendon E, Brunskill SJ, Hyde CJ, Stanworth SJ, Mathur A, Watt SM (2008). Autologous bone marrow stem cells to treat acute myocardial infarction: a systematic review. *Eur. Heart J.*.

[98] Lipinski MJ, Biondi-Zoccai GGL, Abbate A (2007). Impact of intracoronary cell therapy on left ventricular function in the setting of acute myocardial infarction: a collaborative systematic review and meta-analysis of controlled clinical trials. *J. Am. Coll. Cardiol.*.

[99] Huikuri HV, Kervinen K, Niemelä M (2008). [for the FINCELL Investigators]. Effects of intracoronary injection of mononuclear bone marrow cells on left ventricular function, arrhythmia risk profile, and restenosis after thrombolytic therapy of acute myocardial infarction. *Eur. Heart J.*.

[100] Meyer GP, Wollert KC, Lotz J (2006). Intracoronary bone marrow cell transfer after myocardial infarction: eighteen months’ follow-up data from the randomized, controlled BOOST (BOne marrOw transfer to enhance ST-elevation infarct regeneration) trial. *Circulation*.

[101] Leistner DM, Fischer-Rasokat U, Honold J (2011). Transplantation of progenitor cells and regeneration enhancement in acute myocardial infarction (TOPCARE-AMI): final 5-year results suggest long-term safety and efficacy. *Clin. Res. Cardiol.*.

[102] Assmus B, Leistner DM, Schächinger V (2014). Long-term clinical outcome after intracoronary application of bone marrow-derived mononuclear cells for acute myocardial infarction: migratory capacity of administered cells determines event-free survival. *Eur. Heart J.*.

[103] Hirsch A, Nijveldt R, van der Vleuten PA (2011). Intracoronary infusion of mononuclear cells from bone marrow or peripheral blood compared with standard therapy in patients after acute myocardial infarction treated by primary percutaneous coronary intervention: results of the randomized controlled HEBE trial. *Eur. Heart J.*.

[104] Traverse JH, Henry TD, Ellis SG (2011). Effect of intracoronary delivery of autologous bone marrow mononuclear cells to weeks following acute myocardial infarction on left ventricular function: the LateTIME randomized trial. *JAMA*.

[105] Traverse JH, Henry TD, Pepine CJ (2012). Effect of the use and timing of bone marrow mononuclear cell delivery on left ventricular function after acute myocardial infarction: the TIME randomized trial. *JAMA*.

[106] Suerder D, Manka R, Turchetto L (2014). Intracoronary injection of bone marrow derived mononuclear cells, early or late after acute myocardial infarction: long-term effects on global left ventricular function – twelve months MRI and long-term clinical results of the SWISS-AMI trial. *J. Am. Coll. Cardiol.*.

[107] de Jong R, Houtgraaf JH, Samiei S, Boersma E, Duckers HJ (2014). Intracoronary stem cell infusion after acute myocardial infarction. A meta-analysis and update on clinical trials. *Circ. Cardiovasc. Interv.*.

[108] Nowbar AN, Mielewczik M, Karavassilis M (2014). Discrepancies in autologous bone marrow stem cell trials and enhancement of ejection fraction (DAMASCENE): weighted regression and meta-analysis. *BMJ*.

[109] Williams AR, Hare JM (2011). Mesenchymal stem cells: biology, pathophysiology, translational findings, and therapeutic implications for cardiac disease. *Circ. Res.*.

[110] Hare JM, Traverse JH, Henry TD (2009). A randomized, double-blind, placebo-controlled, dose-escalation study of intravenous adult human mesenchymal stem cells (prochymal) after acute myocardial infarction. *J. Am. Coll. Cardiol.*.

[111] Chen SL, Fang WW, Ye F (2004). Effect on left ventricular function of intracoronary transplantation of autologous bone marrow mesenchymal stem cell in patients with acute myocardial infarction. *Am. J. Cardiol.*.

[112] Patel AN, Henry TD, Quyyumi AA (2016). Ixmyelocel-T for patients with ischaemic heart failure: a prospective randomised double-blind trial. *Lancet*.

[113] Chugh AR, Beache GM, Loughran JH (2012). Administration of cardiac stem cells in patients with ischemic cardiomyopathy: the SCIPIO trial: surgical aspects and interim analysis of myocardial function and viability by magnetic resonance. *Circulation*.

[114] The Lancet Editors (2014). Expression of concern: the SCIPIO trial. *Lancet*.

[115] Cross M (2014). Notice of retraction. *Circulation*.

[116] Malliaras K, Makkar RR, Smith RR (2014). Intracoronary cardiosphere-derived cells after myocardial infarction. *J. Am. Coll. Cardiol.*.

[117] Golpanian S, Schulman IH, Ebert RF (2015). Concise review: review and perspective of cell dosage and routes of administration from preclinical and clinical studies of stem cell therapy for heart disease. *Stem Cells Transl. Med.*.

[118] Strauer BE, Steinhoff G, Kipshidze N (2014). Cell therapy for acute myocardial infarction. *Urgent Interventional Therapies*.

[119] Zhu H, Jiang X, Li X (2015). Intramyocardial delivery of VEGF via a novel biodegradable hydrogel induces angiogenesis and improves cardiac function after rat myocardial infarction. *Heart Vessels*.

[120] Kutryk MJB, Stewart DJ, Perin EC, Miller LW, Taylor DA, Willerson JT (2015). Use of gene modified stem cells for acute myocardial infarction. *Stem Cell and Gene Therapy for Cardiovascular Disease*.

[121] Talan MI, Ahmet I, Lakatta EG (2012). Did clinical trials in which erythropoietin failed to reduce acute myocardial infarct size miss a narrow therapeutic window?. *PLoS ONE*.

[122] Roubille F, Prunier F, Barrere-Lemaire S (2013). What is the role of erythropoietin in acute myocardial infarct? Bridging the gap between experimental models and clinical trials. *Cardiovasc. Drugs Ther.*.

[123] Thakker R, Yang P (2014). Mesenchymal stem cell therapy for cardiac repair. *Curr. Treat. Options Cardiovasc. Med.*.

[124] Drummond-Barbosa D (2008). Stem cells, their niches and the systemic environment: an aging network. *Genetics*.

[125] Sahoo S, Losordo DW (2014). Exosomes and cardiac repair after myocardial infarction. *Circ. Res.*.

[126] Singla DK (2016). Stem cells and exosomes in cardiac repair. *Curr. Opin. Pharmacol.*.

[127] Yu B, Kim HW, Gong M (2015). Exosomes secreted from GATA-overexpressing mesenchymal stem cells serve as a reservoir of anti-apoptotic microRNAs for cardioprotection. *Int. J. Cardiol.*.

[128] Khan M, Nickoloff E, Abramova T (2015). Embryonic stem cell-derived exosomes promote endogenous repair mechanisms and enhance cardiac function following myocardial infarction. *Circ. Res.*.

[129] Yang XM, Liu Y, Cui L (2013). Platelet P2Y_12_ blockers confer direct postconditioning-like protection in reperfused rabbit hearts. *J. Cardiovasc. Pharmacol. Ther.*.

[130] Bell RM, Sivaraman V, Kunuthur SP, Cohen MV, Downey JM, Yellon DM (2015). Cardioprotective properties of the platelet P2Y receptor inhibitor, cangrelor: protective in diabetics and reliant upon the presence of blood. *Cardiovasc. Drugs Ther.*.

[131] Marino M, Rizzotti D, Leonardi S (2014). Cangrelor: review of the drug and the CHAMPION programme (including PHOENIX). *Curr. Cardiol. Rep.*.

[132] NCT02313961.

[133] https://www.clinicaltrials.gov/ct2/show/NCT01857414.

[134] NCT02342522.

[135] NCT01938235.

[136] NCT02390674.

[137] NCT01787110.

